# Ethnic-Specific Genetic Association of Variants in the Corticotropin-Releasing Hormone Receptor 1 Gene with Nicotine Dependence

**DOI:** 10.1155/2015/263864

**Published:** 2015-02-22

**Authors:** Xiujun Tang, Shumin Zhan, Liping Yang, Wenyan Cui, Jennie Z. Ma, Thomas J. Payne, Ming D. Li

**Affiliations:** ^1^State Key Laboratory for Diagnosis and Treatment of Infectious Diseases, The First Affiliated Hospital, Collaborative Innovation Center for Diagnosis and Treatment of Infectious Diseases, Zhejiang University School of Medicine, Hangzhou 310009, China; ^2^Department of Public Health Sciences, University of Virginia, Charlottesville, VA 22908, USA; ^3^Department of Otolaryngology and Communicative Sciences, ACT Center for Tobacco Treatment, Education and Research, University of Mississippi Medical Center, Jackson, MS 39213, USA; ^4^Department of Psychiatry and Neurobehavioral Sciences, University of Virginia, Charlottesville, VA 22904, USA

## Abstract

Twin and family studies indicate that smoking addiction is highly influenced by genetic factors. Variants in the corticotropin-releasing hormone receptor 1 (*CRHR1*) gene have been associated with alcoholism and depression. In this study, we tested five single nucleotide polymorphisms (SNPs) in *CRHR1* for their association with ND, which was assessed by smoking quantity (SQ), the Heaviness of Smoking Index (HSI), and the Fagerström test for ND (FTND) in 2,037 subjects from 602 families of either European American (EA) or African American (AA) ancestry. Association analysis of the five SNPs revealed a significant association of rs171440 with SQ in the AA sample and with SQ and FTND in the pooled AA and EA samples. Haplotype-based association analysis indicated significant association of haplotypes C-C (56.9%) and T-C (38.9%), formed by SNPs rs171440 and rs1396862, with SQ in the AA sample, C-C-G (47.6%) with SQ, and T-C-G (42.3%), formed by SNPs rs171440, rs1396862, and rs878886, with SQ and FTND in the pooled AA and EA samples. However, none of these associations remained significant after correction for multiple testing. Together, our results provide suggestive evidence for the involvement of *CRHR1* in ND, which warrants further investigation using larger independent samples.

## 1. Introduction

The world health report in 2002 ranked tobacco smoking as the fourth of the top 10 death risk factors [1]. There are approximately 4,000 chemical compounds and more than 40 types of carcinogens in cigarettes. Tobacco smoking leads to approximately 5,000,000 deaths each year throughout the world; thus, it is a global health problem influencing both smokers and nonsmokers [2].

Nicotine addiction is considered the central problem underlying tobacco smoking [3]. Nicotine enhances excitability and makes an individual smoke repeatedly in order to satisfy physiological and psychological needs and dependence. Once they quit smoking, smokers develop various withdrawal symptoms, including tension, panic, uneasiness, dizziness, and insomnia [4]. Although many smokers want to quit, fewer than 5% succeed, with the complete abstinence rate being 33% at 2 days, 24% at 7 days, 22% at 14 days, 19% at 1 month, 11% at 3 months, 8% at 6 months, and 3% at 6 months after smoking cessation [5].

Numerous studies have focused on the molecular mechanisms underlying nicotine addiction [6–9]. From the neurobiology point of view, nicotine addiction results from repeated long-term exposure to nicotine, leading to central nervous system changes, especially in the midbrain dopamine system, at both the cellular and molecular levels and eventually an addictive state with complex behaviors such as dependence, tolerance, sensitization, and craving [10].

With the advancement of genetics and molecular biology and improvement of technologies, the search for susceptibility loci for ND and its related behaviors has made significant progress in recent years [7, 8, 10, 12]. Data from large twin studies in the US, Scandinavia, Australia, and many other countries demonstrate consistently that genetics impacts the probability of an individual starting smoking and becoming a regular smoker [9, 13, 14]. Epidemiological studies show that there is 47%–76% chance to activate smoking through genetic factors and a 62% chance of continuous smoking [9, 13, 14]. Thus, there exists a strong connection between ND and heredity.

The* CRHR1* gene encodes a G-protein-coupled receptor that binds neuropeptides of the corticotropin-releasing hormone family, major regulators of the hypothalamic-pituitary-adrenal (HPA) axis. The encoded protein is essential for the activation of signal transduction pathways that regulate diverse physiological processes, including stress, reproduction, immune response, and obesity [15, 16]. It has recently reported that variants in* CRHR1* are significantly associated with depression and alcoholism [17–21]. Considering the comorbidity of alcoholism and ND, as well as the strong genetic correlation between the two behaviors [7, 22], we hypothesized that variants in* CRHR1* are also associated with ND.

## 2. Materials and Methods

### 2.1. Subjects and Smoking Phenotypes

The subjects are of EA or AA ancestry and were recruited from the mid-south states of the US, including Mississippi, Tennessee, and Arkansas, during 1999–2004. Comprehensive clinical data are available on each participant, including demographics (e.g., sex, age, race, relationships, weight, height, years of education, and marital status), social environment, the use of other substances, current smoking status and smoking history, medical history, and personality traits. All participants provided informed consent. The study protocol and forms/procedures were approved by the participating institutional review boards.

Three common measures, smoking quantity (SQ), Heaviness of Smoking Index (HSI), and the Fagerstrom test for ND (FTND) [23, 24], were used to assess the ND of each smoker. SQ provides a brief, quantified index of amount of consumption, that is, the number of cigarettes smoked per day. The HSI (0–6-point scale) includes one item addressing quantity (using a 0–3-point compressed format) plus another item assessing smoking urgency, that is, “How soon after you wake up do you smoke your first cigarette?” The FTND (0–10-point scale) includes the HSI plus other indicators of behavioral propensity to smoke in various situations.

The 2037 subjects used in the study were from 602 nuclear families with an average family size of 3.17 ± 0.69 for the EA sample and 3.14 ± 0.75 for the AA sample. The average age was 40.5 ± 15.5 for EAs and 39.4 ± 14.4 for AAs. The average age of smoking onset was 15.5 ± 4.4 for EAs and 17.3 ± 4.7 for AAs. The average FTND score was 6.33 ± 2.22 for EAs and 6.26 ± 2.15 for AAs. The average number of cigarettes smoked per day was 19.5 ± 13.4 for EAs and 19.4 ± 13.3 for AAs. A detailed description of the demographic and clinical characteristics is presented in Table 1 and can be found in other papers reported by this group [25–28].

### 2.2. DNA Samples, SNP Selection, and Genotyping

DNA was extracted from peripheral blood samples using kits from Qiagen Inc. (Valencia, CA, USA). The five SNPs in* CRHR1* were selected from the NCBI dbSNP database on the basis of (1) a preference for SNPs located in the coding or regulatory regions of the gene of interest, (2) high heterozygosity with a minor-allele frequency greater than 0.10, and (3) a relatively uniform coverage of the gene. Information on these SNPs, including location within the gene, chromosomal position, and primer/probe sequences, is summarized in Table 2.

All SNPs were genotyped using the* Taq*Man assay in a 384-well microplate format (Applied Biosystems Inc., Foster City, CA, USA). Briefly, 15 ng of genomic DNA was amplified in a total volume of 7 *μ*L containing an MGB probe and 2.5 *μ*L of* Taq*Man universal PCR master mix. Amplification reaction conditions were 2 min at 50°C and 10 min at 95°C, followed by 40 cycles of 95°C for 25 s and 60°C for 1 min. Allelic discrimination analysis was performed on the Prism 7900HT Sequence Detection System (Applied Biosystems). To ensure the quality of the genotyping, eight SNP-specific control samples were added to each 384-well reaction microplate.

### 2.3. Statistical Analysis

Pair-wise linkage disequilibrium (LD) between SNPs was assessed using the Haploview program [29]. Initially, the pooled AA and EA samples were used to test for possible associations between individual SNPs and ND measures by the Pedigree-Based Association Test (PBAT) statistics (http://www.biostat.harvard.edu/~fbat/pbat.htm) using age, ethnicity, and sex as covariates. For the AA or EA sample, the association of SNPs with ND was tested by the PBAT using age and sex as covariates [30]. Associations between each ND measure and haplotypes containing different combinations of SNPs were determined by the FBAT program [31], with the option of computing* P* values of the* Z* statistics using Monte Carlo sampling under the null distribution of no linkage and no association.

Three genetic models (additive, dominant, and recessive) were tested for both individual and multilocus SNPs (i.e., haplotype analysis) with sex, age, and ethnicity as covariates in the pooled samples and with sex and age as covariates in either AA or EA sample. Three measures, SQ, HSI, and FTND scores, were analyzed individually for each sample. For haplotype-based association analysis, all significant associations were subjected to Bonferroni correction by dividing the significance by the number of major haplotypes (frequency >5.0%).

## 3. Results

### 3.1. Individual SNPs-Based Association Analysis

To determine whether there was any significant association between SNPs and ND measures in the EA or AA sample, we employed the PBAT program under different genetic models (Table 3). We included age, ethnicity, and sex as covariates for the pooled AA and EA samples and age and sex as covariates for each ethnic-specific sample. We found that under the recessive model, SNP rs171440 is significantly associated with SQ (*P* = 0.034) and FTND (*P* = 0.047) in the pooled sample and with SQ (*P* = 0.032) in the AA sample.

### 3.2. Determination of Haplotype Block of SNPs in* CRHR1*

**Figure 1:**
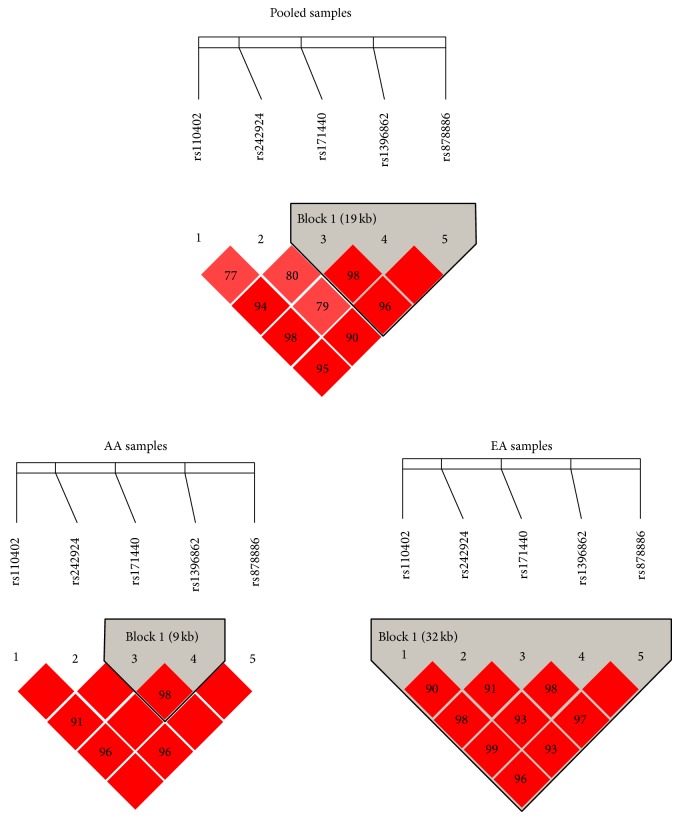
LD structure of five SNPs in* CRHR1* in the pooled, AA, and EA samples. Haplotype block in each sample was defined according to Gabriel et al. [11].

We used the Haploview program to calculate the pair-wise* D*′ values for the five SNPs in* CRHR1* for all the three samples. According to the criteria of Gabriel et al. [11], all five SNPs in* CRHR1* were assigned to an LD block in the EA sample, SNPs rs171440 and rs1396862 to an LD block for the AA sample, and SNPs rs171440, rs1396862, and rs878886 to an LD block for the pooled AA and EA samples (Figure 1). Such LD results indicated a genetic architecture difference in* CRHR1* in the AA and EA samples.

### 3.3. Haplotype-Based Association Analysis

By using the FBAT program, we performed haplotype-based association analysis for the LD block detected in each sample under the three genetic models (Table 4). In the AA sample, we found the haplotype C-C, formed by SNPs rs171440 and rs1396862 with a frequency of 56.9%, showing a significant inverse association with SQ (*Z* = −2.152; *P* = 0.031) under the dominant model (Table 4). Another haplotype, T-C formed by the same SNPs with a frequency of 38.9%, showed a significant association with SQ (*Z* = 2.155; *P* = 0.031) under the recessive model. In the pooled samples, a major haplotype C-C-G (47.6%), formed by SNPs rs171440, rs13968862, and rs878886, showed a significant inverse association with SQ (*Z* = −2.068; *P* = 0.039) under the dominant model (Table 4). Another haplotype, T-C-G (42.3%), showed a significant association with SQ (*Z* = 2.232; *P* = 0.026) and FTND (*Z* = 2.008; *P* = 0.045) under the recessive model. In the EA sample, we found no major haplotype showing significant association with an ND measure (data not shown).

## 4. Discussion

In this study, we revealed significant associations of variants in* CRHR1* with ND in the AA and pooled AA and EA samples at both individual SNP and haplotype level. This conclusion was derived from analyzing 2037 subjects of 602 nuclear families of either AA or EA origin. To correct for the potential impact of age, sex, and ethnicity on our final association results [32], we included age, sex, or ethnicity as covariates in all our analyses. Of the SNPs investigated, only SNP rs171440 showed a significant association with ND in the AA and pooled AA and EA samples. Further, haplotype-based association analysis revealed that haplotypes C-C (56.9%) and T-C (38.9%), formed by SNPs rs171440 and rs1396862 in the AA sample, and haplotypes C-C-G (7.6%) and T-C-G (42.3%), formed by SNPs rs171440, rs1396862, and rs878886 in the pooled AA and EA samples, respectively, are significantly associated with ND.

*CRHR1* is located on chromosome 17q21-22, spanning 20 kb of genomic DNA and containing 14 exons. It belongs to the family of G_s_ protein-coupled receptors [15, 16].* CRHR1* plays a significant role in mediating corticotropin-releasing hormone expression in the HPA axis [33]. Repeated stress exposure increases HPA axis dysregulation and can predict relapse in users of a variety of abused substances, including cocaine, alcohol, nicotine, and opiates, as well as in polysubstance abusers [18–21]. Over the past two decades, preclinical studies have demonstrated consistent effects of* CRHR1* blockade on stress-mediated behavior, related neurochemistry, sympathetic nervous system activation, and neuroendocrine and immune function [34]. Further, variants in* CRHR1* have been associated with depression [35], responses to antidepressants [36], and alcoholism [37]. In the association studies of variants in* CRHR1* with depression [35] and responses to antidepressants [36], three SNPs, namely, rs1876828, rs242939, and rs242941, were investigated, which revealed that the rs242941 G/G genotype and haplotype G-A-G of the three SNPs are associated with fluoxetine therapeutic response in major depressive disorder (MDD) patients with high anxiety. However, a significant association was not observed in MDD patients in the low-anxiety group [36]. In another independent study, a haplotype G-G-T, formed by SNPs rs1876828, rs242939, and rs242941, was significantly overrepresented in MD patients compared with control subjects, which suggests that individuals carrying this haplotype have a greater probability of developing MDD [35].

Additional study has shown the importance of* CRHR1* in integrating gene—environment effects in alcohol use disorders [38]. Association analysis revealed that the SNPs in* CRHR1* and* CRHBP* (corticotropin-releasing hormone binding protein) are associated with blood mRNA concentrations in both alcohol-dependent patients and nondependent controls [39]. These findings imply that carriers of a homozygous T allele of rs110402 in* CRHR1* combined with a homozygous G allele of rs3811939 in* CRHBP* are more than twice as likely to develop comorbid alcoholic use disorders (AUD) than are carriers of all other possible genotype combinations. This suggests that a particular combined genotype of rs110402 in* CRHR1* and rs3811939 in* CRHBP* plays an important role in alcoholism through gene-by-gene interaction. Together, these findings provide evidence for the involvement of* CRHR1* in alcoholism risk through interactions with other genes.

The findings on ND reported here are consistent with the role of* CRHR1* in the initiation and maintenance of alcoholism [40]. In contrast to the extensive research effort to understand the genetic contribution to alcoholism risk, there has been no research directed at understanding the genetic influence of* CRHR1* on smoking behavior. Thus, this study represents the first investigation of the role of* CRHR1* in the etiology of smoking and demonstrates a significant association of* CRHR1* with ND.

These results should be interpreted in light of the strengths and limitations of the study. The strengths include well-ascertained smoking-related phenotypes, relatively large family-based samples, and relatively homogeneous samples as they were all recruited from the mid-south states of the US. Importantly, because our samples are family based, they could minimize or eliminate potential confounding effects of population stratification as an explanation for the different results in the two ethnic groups. On the other hand, there are several limitations of this study. For example, the sizes of the AA and EA samples are not equal, the AA sample being much larger, which may explain why we failed to detect any significant association of variants in* CRHR1* with ND in the EA sample. Second, our study could not account for the potential contribution of environmental factors such as the influence of friends or relatives on each smoking behavior. Third, the association of variants in* CRHR1* with ND that we detected is relatively weak; if we applied Bonferroni correction to our thresholds for *P* values, all detected associations became nonsignificant. Finally, only a limited number of SNPs were genotyped in this study. Thus, the conclusions drawn from this study should be treated with caution, and more independent replications with more SNPs genotyped for each sample, ideally in larger and equal samples, are needed.

In summary, we are the first to reveal a significant association of variants in* CRHR1* with ND. We identified not only individual SNPs but also haplotypes that are significantly associated with ND in the AA and pooled AA and EA samples. These findings indicate that* CRHR1* represents a plausible candidate gene for involvement in ND, and more association analyses using independent samples are warranted.

## Figures and Tables

**Table 1:** Clinical characteristics of pooled, EA, and AA samples (mean ± SD where appropriate).

Characteristic	EA	AA	Pooled populations
Number of families	200	402	602
Avg. members/family	3.17 ± 0.69	3.14 ± 0.75	3.15 ± 0.73
Number of subjects	671	1,366	2,037
% female	69.5	66.1	67.2
Age (years)	40.5 ± 15.5	39.4 ± 14.4	39.7 ± 14.8
Number of smokers	515	1,053	1,568
Years smoked	23.2 ± 13.5	20.4 ± 12.5	21.3 ± 12.9
Cigarettes smoked/day	19.5 ± 13.4	19.4 ± 13.3	19.5 ± 13.3
FTND score	6.33 ± 2.22	6.26 ± 2.15	6.29 ± 2.17
Age of smoking onset (yrs)	15.5 ± 4.4	17.3 ± 4.7	16.7 ± 4.7

**Table 2:** Positions, nucleotide variation, minor allele frequency, and primer/probe sequences of five SNPs in *CRHR1*.

dbSNP ID	SNP location	Chrom. position	Alleles	Minor allele frequency^*^	Forward (F) and reverse (R) primer and probe (P) sequences
rs110402	5′ UTR	41235818	C/T	0.44	F: TGATGGTTCACACAGGCATTTTCTA R: GAGGAAAGGTTGGTTTGGGAATTTT P: CTTTGCATAACG/ACAACAC

rs242924	Intron 1	41241147	C/A	0.41	F: ACAAGCCTTCCAAAGACACTCA R: GGCATGGCTGCTGCTG P: ACCCTCTA/CCATTTTT

rs171440	Intron 1	41249267	C/T	0.47	F: TCTGGTCCCCTGCTCTGTAG R: GCTCCAGGCTGTCACCAT P: CCAAGAGAAG/ATGTCCTT

rs1396862	Intron 3	41258778	T/C	0.10	C_7450777_10

rs878886	3′ UTR	41268271	G/C	0.25	F: CCTTCTCCCAGAGCACAAGA R: CCTCCCCACGGTTGCC P: CCCCAGGG/CCCCAGT

^*^From http://www.lifetechnologies.com/.

**Table 3:** *P* values for association of NSPs in *CRHR1* with three ND measures in the pooled, African American and European American samples.

dbSNP ID	Pooled samples			AA sample			EA sample		
	SQ	HSI	FTND	SQ	HSI	FTND	SQ	HSI	FTND
rs110402	0.191	0.592	0.268	0.087	0.301	0.263	0.904	0.717	0.453
rs242924	0.296	0.550	0.289	0.255	0.467	0.503	0.888	0.879	0.342
rs171440	0.034^r∗^	0.069	0.047^r^	0.032^r^	0.080	0.103	0.503	0.372	0.162
rs1396862	0.420	0.555	0.285	0.174	0.156	0.148	0.663	0.756	0.545
rs878886	0.612	0.428	0.204	0.175	0.156	0.148	0.422	0.316	0.277

^*^Superscripts indicate the genetic models used for analysis; r: recessive model.

**Table 4:** *Z* and permutated *P* values for association of major *CRHR1* haplotypes formed by SNPs rs171440, rs1396862, and rs878886 with three ND measures in the pooled AA and EA samples and SNPs rs171440 and rs1396862 in the AA sample.

Sample	SNPs	Haplotype	Frequency (%)	SQ	HSI	FTND
AA	rs171440 and rs1396862	C-C	56.9	−2.152^d^ (0.031)	−1.416 (0.157)	−1.047 (0.295)
		T-C	38.9	2.155^r^ (0.031)	1.619 (0.105)	1.467 (0.142)

Pooled samples	rs171440, rs1396862, and rs878886	C-C-G	47.6	−2.068^d∗^ (0.039)	−1.334 (0.182)	−1.201 (0.230)
		T-C-G	42.3	2.232^r^ (0.026)	1.840 (0.066)	2.008^r^ (0.045)
		C-T-C	8.2	−0.315 (0.753)	−0.496 (0.620)	−1.195 (0.232)

^*^Superscripts indicate the genetic models used for analysis; d: dominant; r: recessive model. Except for the model indicted, all other *P* values shown in the Tables 3 and 4 were calculated under the additive model.
